# Study on the grain refinement mechanism of Mg-Al alloy based on carbon addition

**DOI:** 10.1371/journal.pone.0271583

**Published:** 2022-08-04

**Authors:** Lifeng Sun, Zhongyu Gao, Zhongchao Hu, Huyan Chen, Jianwen Cai, Xiaoou Cai

**Affiliations:** 1 Emergency Research Institute, China Coal Research Institute CCRI, Beijing, China; 2 State Key Laboratory of Efficient Mining and Clean Utilization of Coal Resources, Beijing, China; 3 Foshan Polytechnic, Foshan, GuangDong, China; College of Engineering, University of Saskatchewan, CANADA

## Abstract

In this study, a comprehensive treatment process based on the rotary injection of Ar+CO_2_ Mg-Al alloy melt is proposed. The effect of carbon on the grain refinement of Mg-Al alloy is studied according to the proposed integrated treatment process. The regularity of carbon refinement in the Mg-Al alloy is examined by microstructural observation and theoretical calculation. The results show that carbon has no effect on the grain refinement of Mg-Al alloy when the Al content is less than 1wt.%. However, when the Al content reaches 2 wt.%, the refining effect is obvious, and the grain refinement efficiency is 62%. The refining effect increases with the increase in the Al content, and the refinement efficiency becomes 79% when the Al content reaches 9 wt.%. The size of Al-C-O in the matrix is approximately 5μm, which confirms the existence of Al_4_C_3_ phase exists as a heterogeneous nucleating agent. The theoretical calculations suggest that the Al_4_C_3_ heterogeneous nucleating agent cannot be formed when the Al content in the Mg alloy is less than 1.34%, so there is no thinning effect under such Al content. The crystallographic calculations reveal that the mismatch between the Al_4_C_3_ phase and Mg alloy matrix is only 4.05%, and Al_4_C_3_ can exist as a heterogeneous nucleating agent for α-Mg phase. Combining the measured solidification curves with the classical nucleation theory, the wetting angle of Mg-Al alloy on Al_4_C_3_ is calculated to be 24.3°.

## 1. Introduction

As the lightest structural metal, Mg has the characteristics of high specific strength, high specific stiffness, and outstanding casting performance [1–3]. Consequently, Mg alloys are extensively used in many fields, such as aerospace, transportation, and 3C industries [4, 5]. However, the low ductility, difficult deformation, and easy cracking hinder the wider applications of Mg alloys [6–8]. Refinement treatment can improve the plastic deformation ability of these alloys, and superplasticity can be realized when the grain size is reduced to a certain extent. Grain refinement is also of great significance to improve the performance of Mg alloy castings. The refinement treatment causes the primary α-Mg crystal to change from coarse dendrites to fine equiaxed crystals, and in this process, the distribution of second phase particles in the alloy becomes more uniform. At the same time, this treatment can improve the strength, and elongation and reduce the casting segregation. The existing refinement methods of Mg-Al alloys mainly include carbon inoculation method, superheating method, anhydrous FeCl_3_ method, deformation treatment method, alloying method, etc [9–11]. The addition of Fe element in the anhydrous FeCl_3_ method reduces the corrosion resistance of the alloys, so it is not widely used in industrial applications [12]. Due to the high temperature involved in the superheating treatment, the Fe content in the melt increases, the liquid Mg oxidizes, and the suction becomes severe. Further, the equipment requirements for this method are too high. Therefore, it is gradually being replaced by other methods in industrial production [13]. In the alloying methods, the alkali metals Sr, Ca and rare earth elements are usually added to refine the Mg-Al alloys [14–16]. The generally considered refinement mechanism is that the addition of alloying elements forms high melting point substances that can be used as heterogeneous nucleating particles. Another view is that the addition of alloying elements at the front of the solid/liquid interface causes the composition to be supercooled, which can stimulate more nucleating particles. The third mesoscopic view is that the addition of alloying elements leads to the occurrence of precipitation in front of the solid-liquid interface, which hinders the growth of grains. Therefore, the mechanism of refining Mg-Al alloy by alloying method needs to be further studied. Carbonaceous inoculation method has the advantages of obvious refinement effect, low operation temperature, convenient operation, and less fading. Consequently, it is considered to be the most effective refinement method with immense development prospect [17]. The proposed grain refinement mechanisms of carbonaceous inoculation method, include solubility theory, Al_4_C_3_ heterogeneous nucleation theory, Al-C-O compound nucleation theory, carbon segregation theory, and dual nucleation theory [18–21].

Zhang et al. [19] established the edge to edge matching model, which supported the nucleation theory of Al_4_C_3_ heterogeneous nucleus to a certain extent. Xi et al. [22] summarized the grain refinement mechanisms of carbon inoculation, for Mg-Al alloy and presented the limitations of existing explanations as well as the future research directions. Liu et al. [23] developed a new Al_4_C_3_-SiC/Al master alloy for the grain refinement of Mg-Al alloy and investigated its refining properties and refinement mechanism. Saha and Ravindran [24] examined the effect of ZnO on the microstructure and mechanical properties of permanent magnet mold cast AZ91E Mg alloy for improving the Mg grain refinement. Kim et al. [25] found that C_2_Cl_6_ had an obvious effect on the refinement of Mg-Al alloy containing Mn, but they failed to provide sufficient microscopic analysis evidence for Al_4_C_3_ particles acting as the nucleating substrate for Al_8_Mn_5_ phase. Cao et al. [26] suggested that both Fe and Mn played a vital role in refining Mg-Al alloy by superheating method. With the increase in the Fe content, the refining effect was enhanced, but excessive Mn content could lead to grain coarsening. According to the above analysis, the existing studies on the refinement of Mg-Al alloy crystals mainly focus on the impact analysis of the new grain refiners, but the new grain refiners are operate under a specific environment, mostly in the aspects of lattice mismatch, chemical reaction thermodynamic calculation, and so on.

Clarifying the influence and relevant mechanism of carbon on the grain refinement of Mg-Al alloy is of great significance to improve the properties of Mg alloy castings. The refinement treatment, which changes the primary α-Mg crystal from coarse dendrites to fine equiaxed crystal, can simultaneously improve the strength and elongation and reduce the casting segregation [27]. Grain refinement makes the second phase particles on the grain boundary to becomes fine and diffused, and it is easier to diffuse into the matrix during heat treatment, so the heat treatment efficiency of the alloy is obviously improved [28]. In addition, grain refinement can improve the corrosion properties of the alloy [29]. This study mainly focuses on analyzing the grain refinement mechanism of carbon on Mg-Al alloy. The Ar+CO_2_ gas is used as the refining agent, which is injected into the Mg-Al alloy melt for degassing the alloy. At the same time, carbon is introduced into the alloy to refine the grains. The refinement mechanism is comprehensively examined, which potentially lays a foundation for the development of green and pollution-free, carbon-containing grain refiner.

## 2. Test materials and methods

### 2.1 Test materials

AZ91 alloy was used as the raw material. Its specific composition is shown in Table 1. The Al content of the Mg-Al alloys was adjusted by adding pure Mg, while Mg-Mn alloy and Zn elements were added to ensure the same contents of Mn and Zn elements in the original AZ91 alloy.

**Table 1:** Composition of AZ91 alloy.

Element	Al	Zn	Mn	Fe	Ni	Mg
**Mass fraction (wt.%)**	8.91	0.61	0.20	0.0010	0.00080	Allowance

### 2.2 Test methods

The refinement method involved the rotary injection of Ar+CO_2_ gas into the melt of Mg-Al alloy melt, for degassing the alloy, and C was also introduced into the alloy to refine the grains. The melt was poured into a sand mold with a diameter of 45 mm and a height of 100 mm, and the samples were taken 40 mm above the bottom surface. The metallographic sample with dimensions of 15×15×10 mm^3^ was prepared and etched with nital (solution of nitric acid and alcohol) after polishing. The microstructure of the alloy was examined by optical microscopy (OM) and scanning electron microscopy (SEM; Hitachi-4700) in conjunction with energy dispersive X-ray spectroscopy (EDS). The grain size of the alloy was measured by the linear intercept method. The thermal parameters of the alloy were evaluated by combining differential thermal analysis (DTA) with thermogravimetry (TG) technology (TG/DTA6300 thermal analyzer).

**Fig 1:**
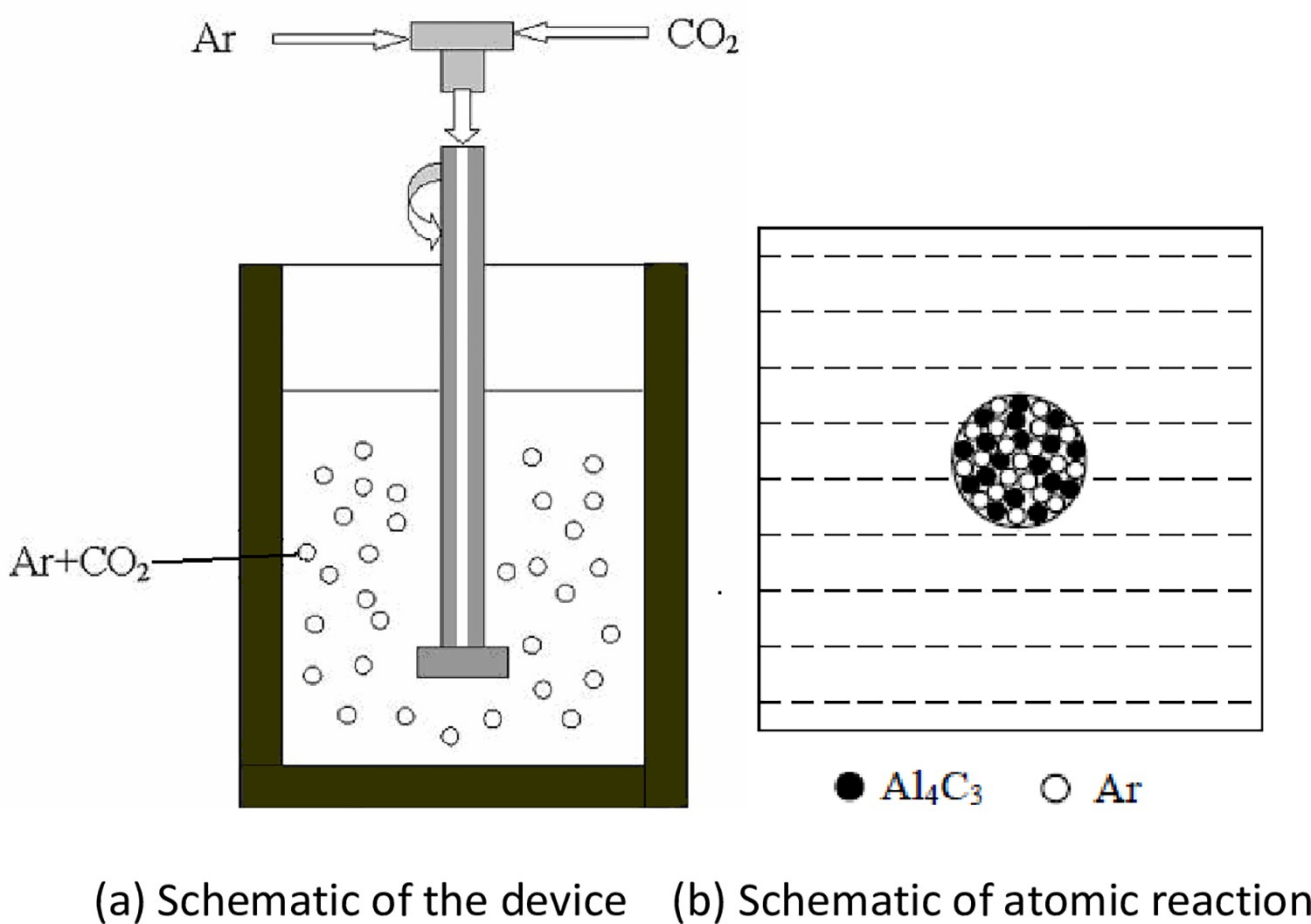
Schematic of refinement principle based on the rotary injection of Ar+CO_2_. (a) Schematic of the device; (b) Schematic of atomic reaction.

A schematic of the rotary injection technology is shown in in Fig 1, where CO_2_ gas is introduced into the AZ91 alloy melt with Ar as the carrier. Ar and CO_2_ are mixed after passing through two flow meters, and the mixed gas is introduced into an air storage tank for uniform mixing with a rotation speed of 280 rpm and total flow rate of 1000 ml/min. When the total flow is constant, the addition amount of C per unit time is controlled by adjusting the flow of CO_2_.

## 3. Impact of C on refinement of Mg-Al alloy

**Fig 2:**
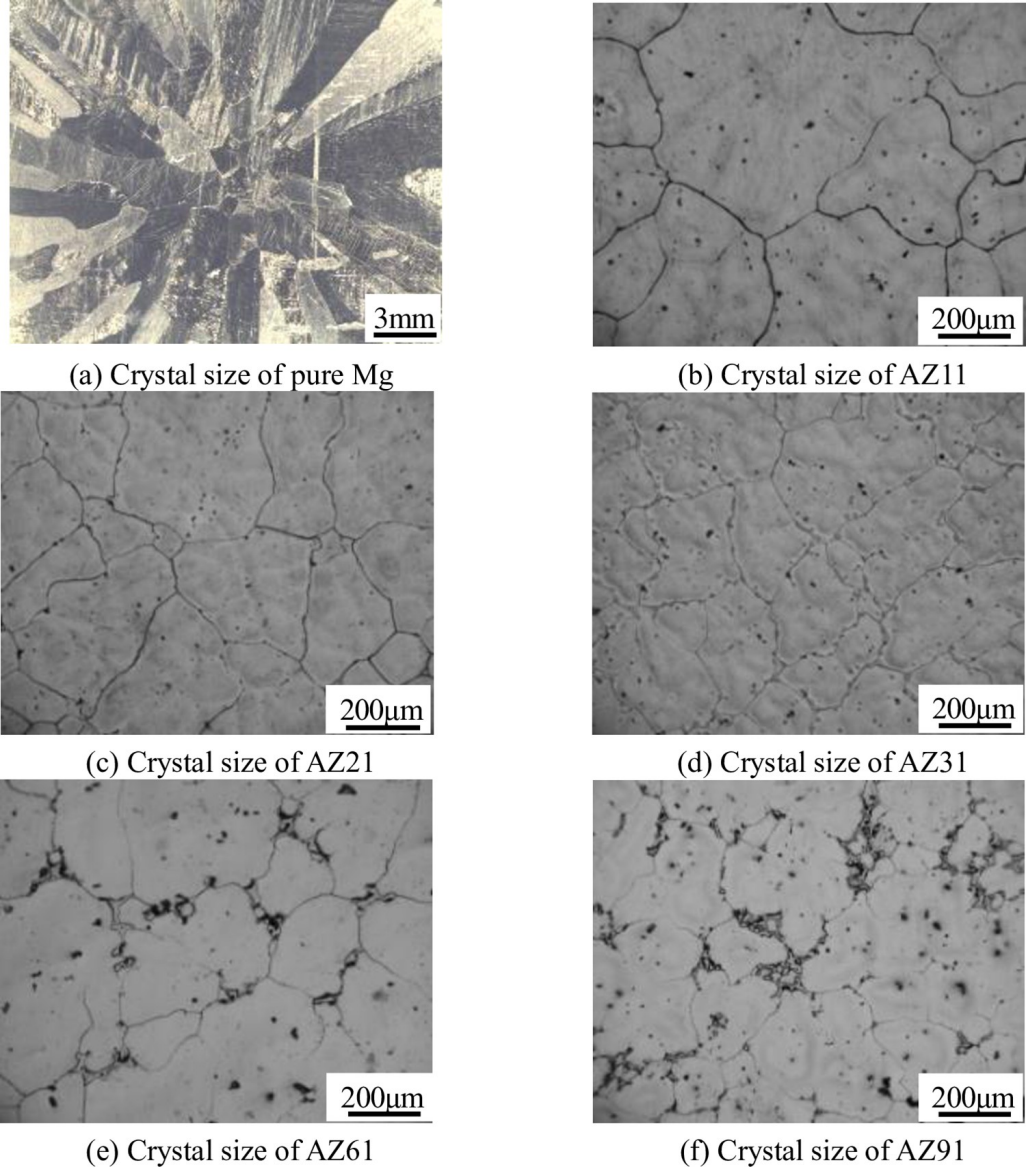
Microstructure of Mg-Al alloys with different Al contents without refinement. (a) Crystal size of pure Mg; (b) Crystal size of AZ11; (c) Crystal size of AZ21; (d) Crystal size of AZ31; (e) Crystal size of AZ61; (f) Crystal size of AZ91.

**Fig 3:**
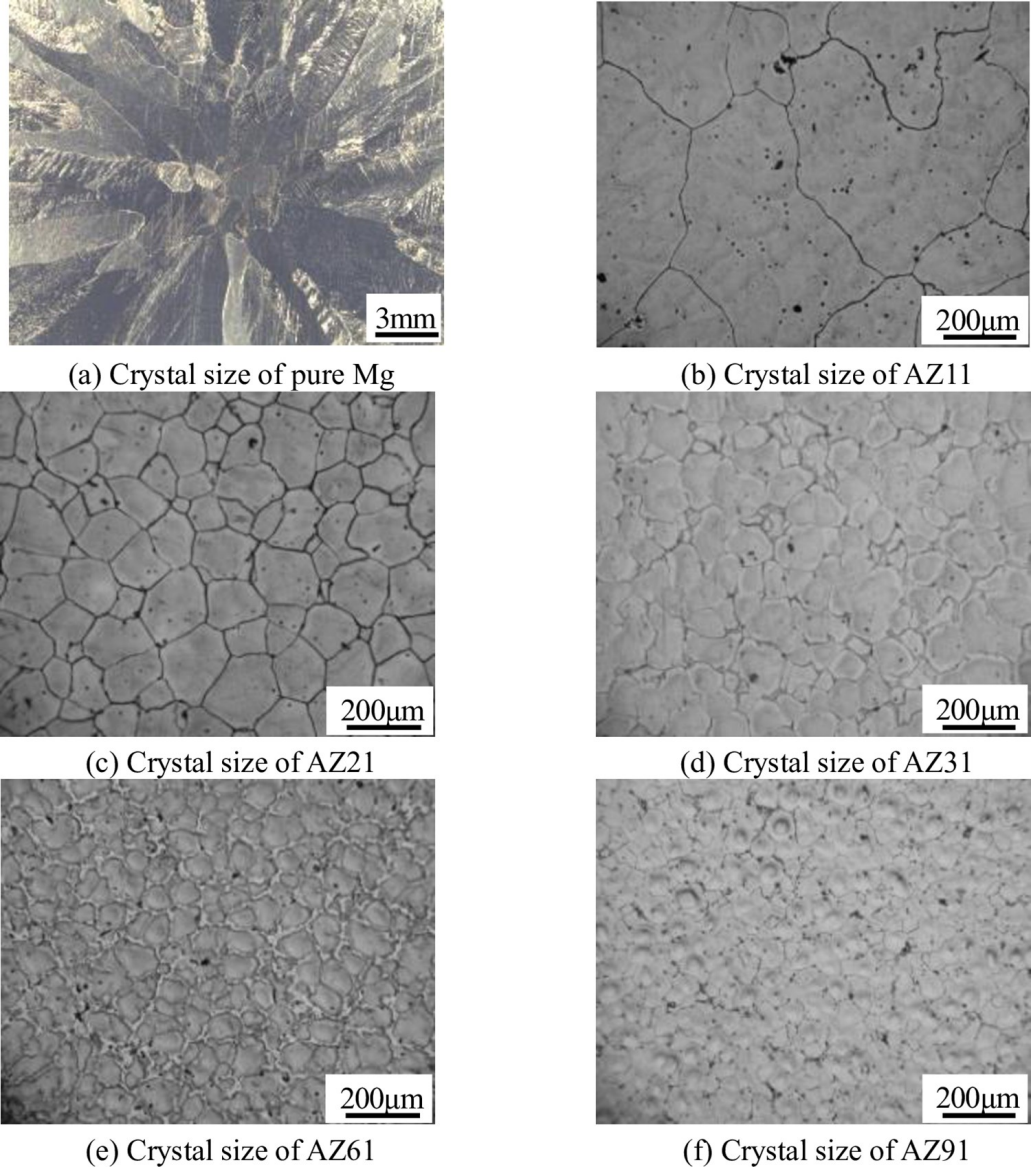
Metallographic analysis of Mg-Al alloys after refinement. (a) Crystal size of pure Mg; (b) Crystal size of AZ11; (c) Crystal size of AZ21; (d) Crystal size of AZ31; (e) Crystal size of AZ61; (f) Crystal size of AZ91.

The refining effect of C on pure Mg, AZ11(containing 1 wt.% Al), AZ21(containing 2 wt.% Al), AZ31(containing 3 wt.% Al), AZ61(containing 6 wt.% Al), and AZ91 (containing 9 wt.% Al) alloys are shown in Figs 2 and 3. It is clear seen from Fig 2 that the grain size of Mg-Al alloy decreases with the increase in the Al content. This is because Al inhibits the grain growth in Mg, which refines the α-Mg matrix. However, when the Al content is less than 3 wt.%, the effect of Al on the refinement of Mg alloy is obvious. When the Al content is greater than 3 wt.%, the grain size of the alloy does not change significantly.

Fig 3 shows the metallographic structure of Mg-Al alloys with different Al contents refined by adding C. Compared with Fig 2, it can be seen that C has no obvious refining effect on the pure Mg and Mg-Al alloy when the Al content is 1 wt.%. With the increase in the Al content, the refining effect becomes more and more increasingly obvious.

To better understand the thinning effect of C on the Mg-Al alloys with different Al contents, the thinning efficiency *ω* is defined as follows:

$$\omega =(1-\frac{g_{f}}{g_{nf}})100\%$$ (1)

where *g*_*f*_ is the grain size after refinement (μm), and *g*_*nf*_ is grain size before refinement (μm).

**Fig 4:**
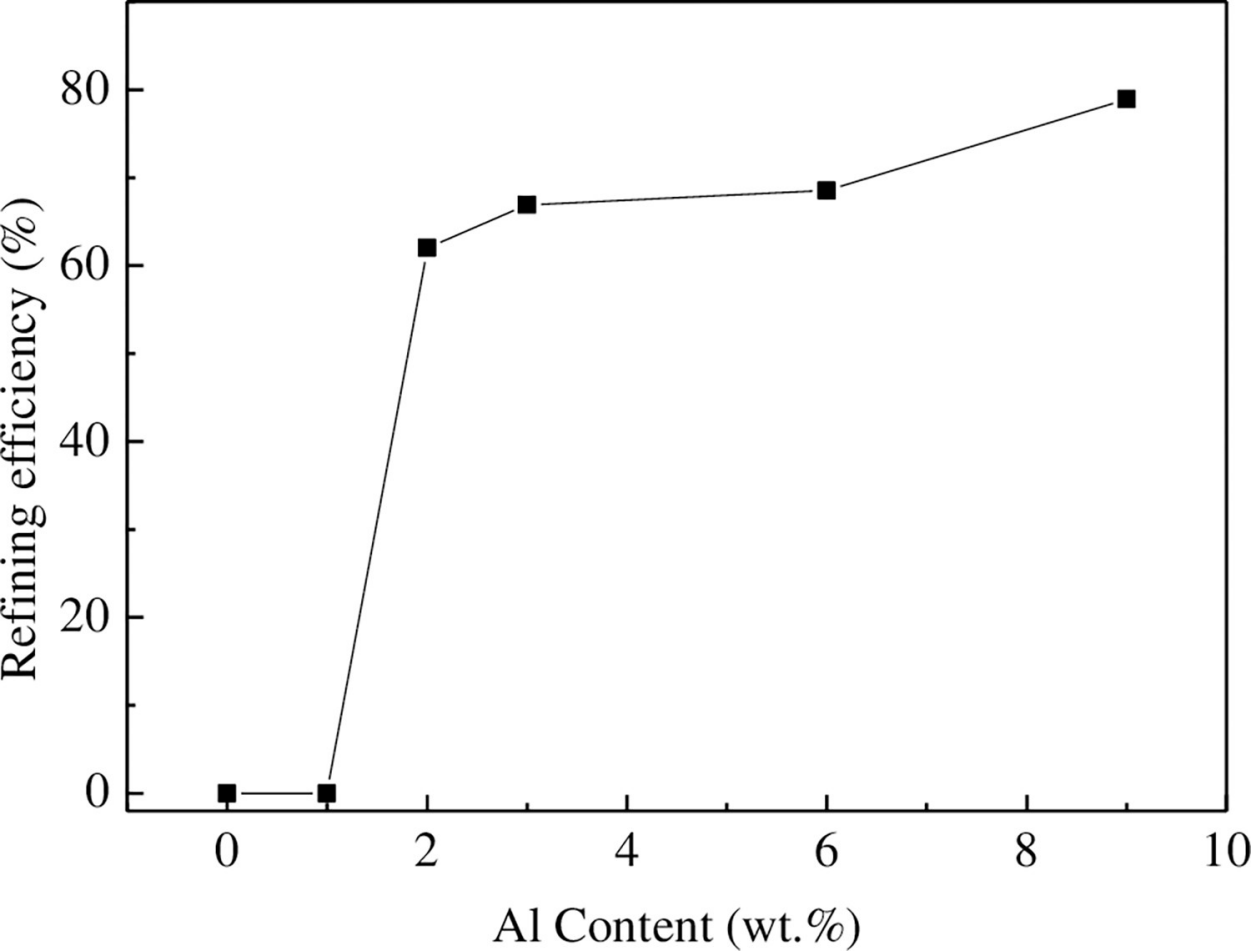
Refinement efficiency of Ar+CO_2_ in Mg-Al alloys with different Al contents.

The grain size obtained from Figs 2 and 3 are substituted into Eq (1) to examine the influence of Al content on the refinement effect, as shown in Fig 4 (**S1 Table in S1 File**). It is evident that when the Al content is less than 1 wt.%, C has no thinning effect on the Mg-Al alloy. When the Al content is 2 wt.%, the refinement efficiency is 62%, and the refinement effect of C on Mg-Al alloy is very obvious. Further, the refinement effect increases with the increase in the Al content. When the Al content reaches 9 wt.%, the refinement efficiency becomes 79%.

## 4. Refined microstructure and properties

### 4.1 Microscopic observation

**Fig 5:**
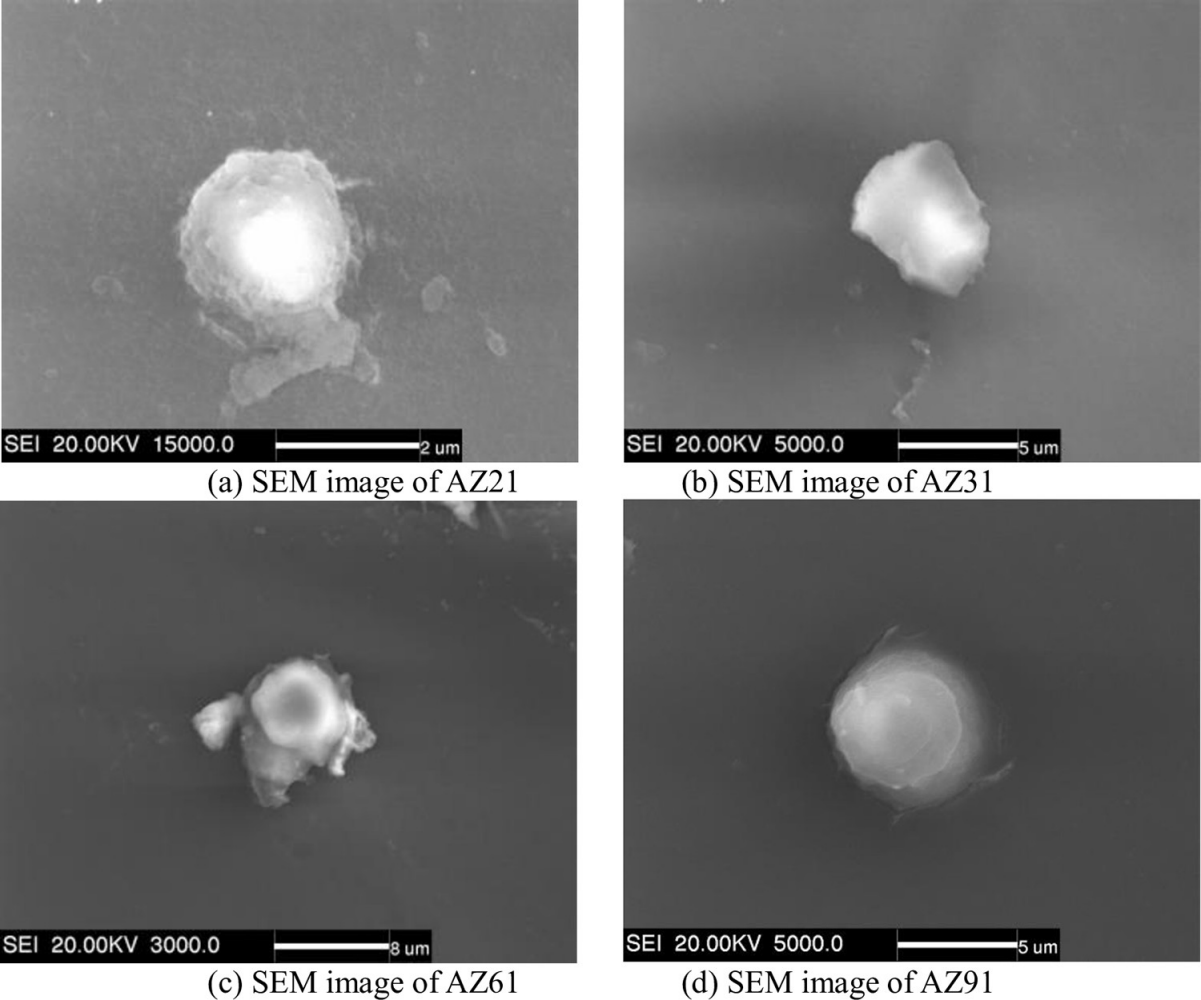
SEM image of Mg-Al alloys with added Al_4_C_3_. (a) SEM image of AZ21; (b) SEM image of AZ31; (c) SEM image of AZ61; (d) SEM image of AZ91.

AZ11, AZ21, AZ31, AZ61, and AZ91 alloys were refined by introducing C, and the microstructure after refinement and solidification was observed. Fig 5 shows the microstructure of the alloy after refinement. It can be seen that there are spherical particles in the grains of AZ21, AZ31, AZ61 and AZ91 alloys with a diameter of approximately 2–5 μm. No spherical particles are found in the AZ11 alloy. This is because when the Al content is less than 1 wt.%, the thermodynamic condition for generating heterogeneous nucleating particles is not satisfied, so no nucleating particles are observed. Therefore, when the Al content is less than 1 wt.%, the introduction of C has no refining effect. It can also be seen from Fig 4 that the refinement efficiency of C addition becomes obvious with the increase in the Al content, and there is no refinement effect when the Al content is less than 1 wt.%. When the Al content reaches 2 wt.%, particles with a diameter of nearly 2 μm are observed in the microstructure, and the alloy is composed of Al, C, O ternary compound. When the Al content reaches 3 wt.%, the particle size increases to approximately 5 μm, and with the continuous increase in the Al content, the particle size does not increase and remains constant at nearly 5 μm.

**Fig 6:**
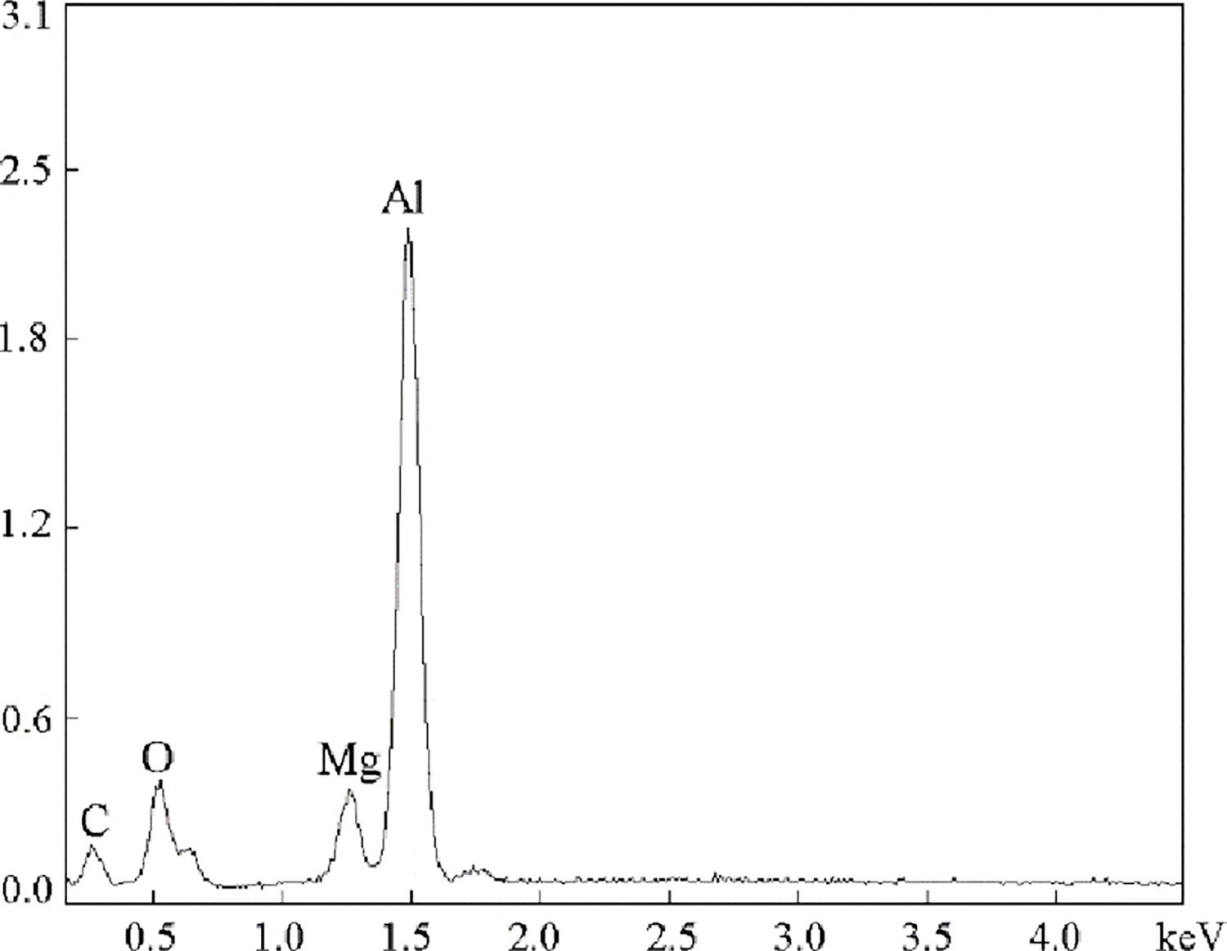
Microstructural energy spectrum analysis of AZ91 alloy after particle refinement.

The energy spectrum in Fig 6 shows that there are Al, C and O ternary compounds in the matrix of refined AZ91 alloy. The size of the phase is approximately 5μm, which is consistent with the size of heterogeneous nucleating particles. The reaction of C with Al in the alloy melt is expressed as follows:

$$3C+4Al\to Al_{4}C_{3}$$ (2)

Therefore, it can be inferred that the Al_4_C_3_ particles in the Mg-Al alloy act as heterogeneous nucleating particles to refine the grains, which is consistent with the previous reports [20, 30, 31]. The existence of O in the microstructure may be attributed to the hydrolysis of Al_4_C_3_ during the sample preparation process [32], i.e.,

$$Al_{4}C_{3}(s)+12H_{2}O(1)\to 4Al{\left(OH\right)}_{3}(s)+3CH_{4}(g)$$ (3)

**Fig 7:**
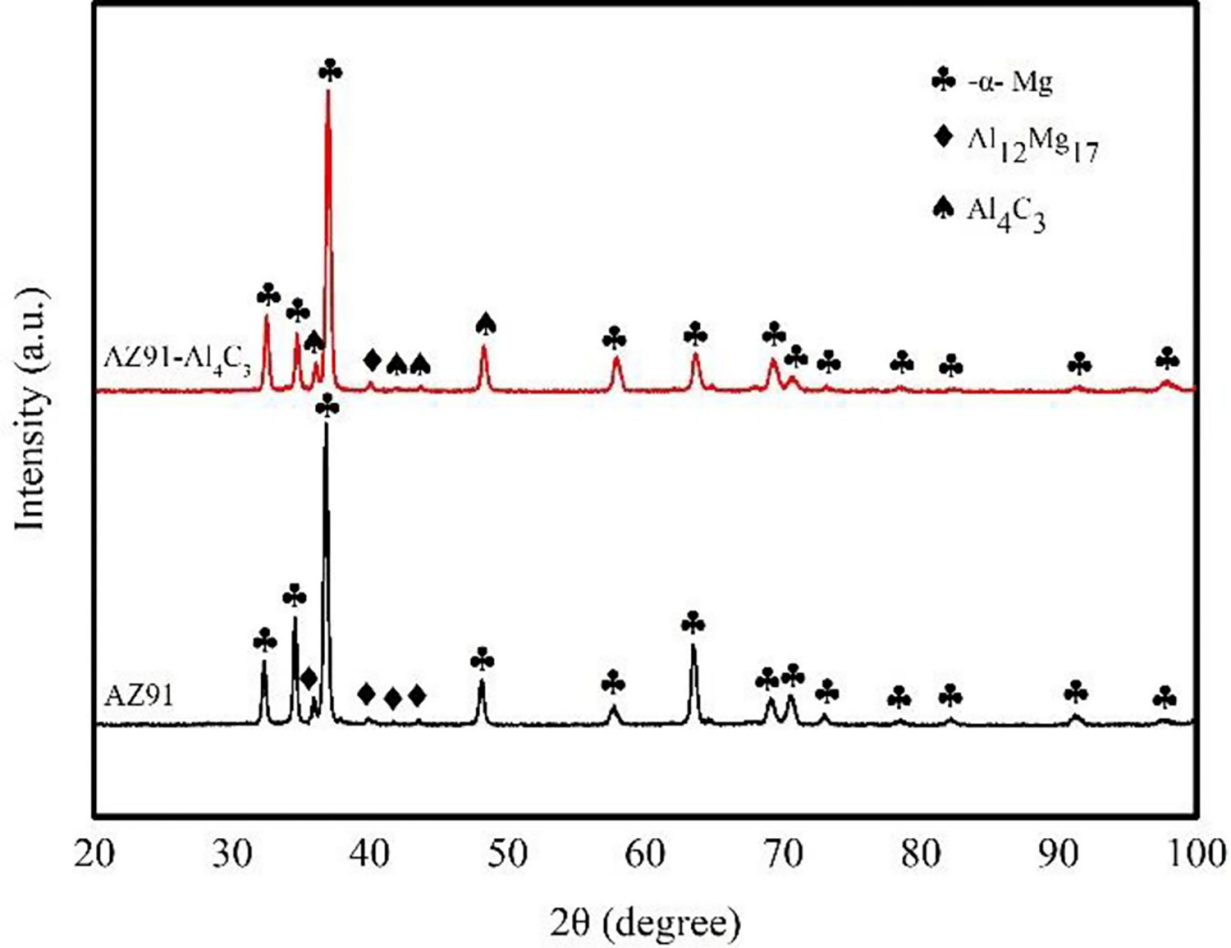
XRD analysis of AZ91 alloy.

XRD analysis was made for AZ91 alloy, as shown in Fig 7. It can be seen from the figure that only α-Mg and Al_12_Mg_17_ phases, and after adding "C" to AZ91 alloy, in addition to the above two phases, there is Al_4_C_3_ phase. In combination with the microstructure in Fig 5, the energy spectrum analysis in Fig 6 and the reaction equation in Eq (2), it is also confirmed that Al_4_C_3_ can be used as heteromorphic nucleoplasm of α-Mg.

**Fig 8:**
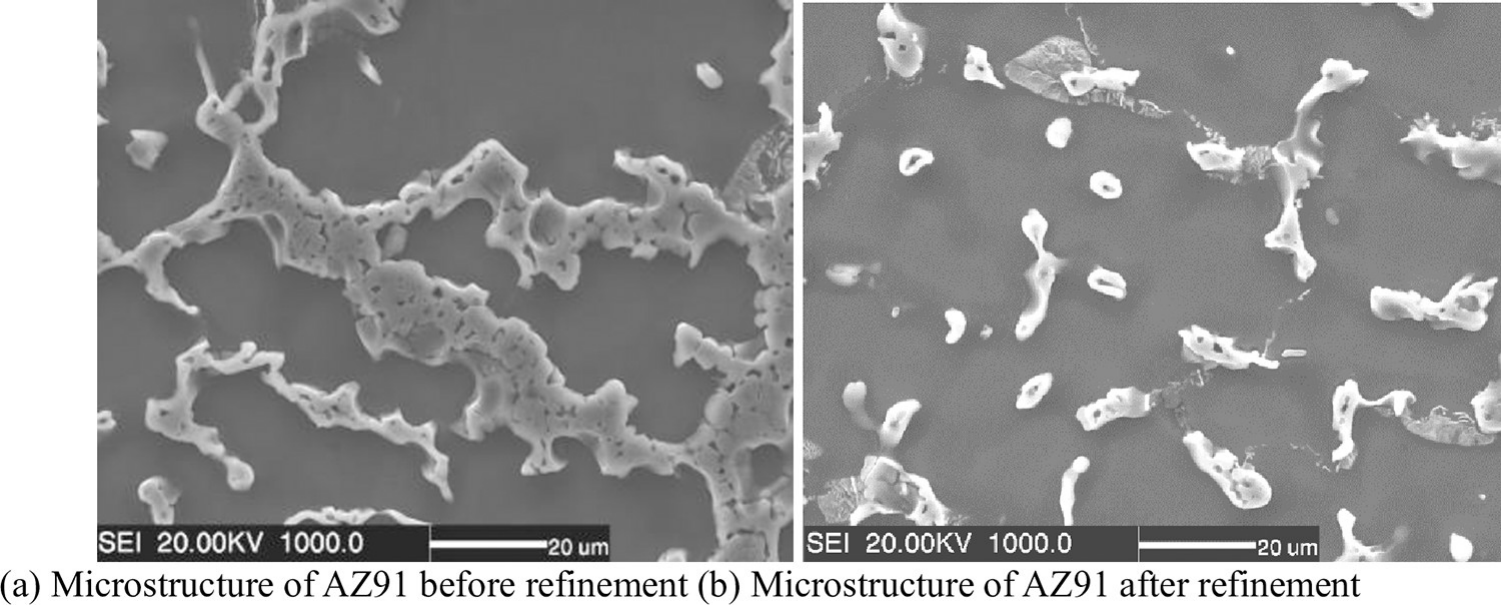
Microstructure of AZ91 before and after refinement. (a) Microstructure of AZ91 before refinement; (b) Microstructure of AZ91 after refinement.

Refinement only has a certain effect on the morphology of the second phase of Mg-Al alloy, so only the microstructure of AZ91 alloy before and after refinement is examined in this study, as shown in Fig 8. Fig 8(A) shows the microstructure of AZ91 before refinement, and the newly-formed α-phase of AZ91 alloy precipitates and grows first. The volume fraction of the α-phase is larger than that of eutectic liquid phase before eutectic composition is reached. At the beginning of the transition, the eutectic α-phase is attached to the newly-formed α-phase, and the eutectic β-Mg_17_Al_12_ phase grows independently at the grain boundary. With the growth of β-phase, the depletion of Al atom leads to an increase in the concentration of Mg in the remaining melt. At this time, the diffusion of Mg is limited by the grown β-phase, and it cannot attach to the newly-formed α-phase but can only nucleate near the β-phase. Finally, the eutectic α-phase exists in the middle of the β-phase as an island. When β-phase grows to a certain extent, another kind of lamellar eutectic structure is formed. The interdiffusion of Mg atoms within the α-phase becomes relatively difficult due to the increase in the diffusion distance, and the α-phase also attaches to the side of β-phase and grows alternately with β-phase, forming lamellar eutectic structure α+β.

Fig 8(B) shows the microstructure of AZ91 alloy after refinement. It can be seen that when the refiner is added, the β-phase particles become dispersed and fine, and a part of the dissociated eutectic morphology is still characterized by the presence of eutectic α-Mg phase in the β phase, which is island-like, and there are still many lamellar eutectic structures.

### 4.2 Mechanical properties

The mechanical properties of the alloy (T6 temper) at room temperature are shown in Table 2. It can be seen that the AZ91 alloy exhibits obvious improvement in the tensile strength, yield strength, and elongation after refinement treatment. Specifically, under the condition of metal mold casting, the tensile strength, yield strength and elongation of the refined AZ91 alloy are increased by approximately 11%, 15% and 22%, respectively, with respect to those of the unrefined alloy. As mentioned before, this improvement can be attributed to the fact that the refinement eliminates the coarse dendritic structure and forms fine equiaxed crystals. According to the grain boundary strengthening theory, the thinner the grain boundary, the larger the area of the grain boundary, and the greater the hindrance effect of the grain boundary on the intragranular dislocation, the higher the external force required to deform the grains, and the greater the strength of the alloy. In addition, the grain refinement changes the morphology of the intergranular Mg_17_Al_12_ from coarsened to fine and dispersed, which increases the effect of dispersion strengthening of the alloy.

**Table 2:** Effect of refinement on the mechanical properties of AZ91 alloy.

Alloy state	Mechanical properties (T6 temper)		
	Tensile strength	Yield strength	Elongation
	(MPa)	(MPa)	(%)
**Before refinement**	229.8	116.7	5.75
**After refinement**	206.3	101.6	4.72

## 5. Refinement mechanism

The thermodynamic analysis of the formation of Al_4_C_3_ in the Mg-Al alloy, crystallographic conditions of heterogeneous nucleation, and the wetting angle of α-Mg on Al_4_C_3_ show that Al_4_C_3_ can be used as an effective heterogeneous nucleation substrate for α-Mg.

### 5.1 Thermodynamic conditions for the formation of Al_4_C_3_

It can be seen from the above analysis that C affects the grain refinement of Mg-Al alloy only when the Al content is greater than 2 wt.%. The refinement mechanism is that Al_4_C_3_ heterogeneous nucleating particles are generated in Mg-Al alloy melt. In other words, no Al_4_C_3_ is formed in the Mg-Al alloy melt with the Al content less than 2 wt.%. Therefore, Al_4_C_3_ is generated during the refinement process of Mg-Al alloy when the necessary thermodynamic conditions are satisfied. When C forms Al_4_C_3_ in the melt of Mg-Al alloy, the Gibbs free energy required for the formation of intermetallic compounds is expressed as follows [26]:

$$\Delta G^{0}=-266266+96.14T+RT\ln\frac{a_{Al_{4}C_{3}}}{a_{Al}^{4}a_{C}^{3}}$$ (4)

where $a_{Al_{4}C_{3}}$ is activity of Al_4_C_3_ particle in Mg-Al alloy melt, *a*_*C*_ is activity of C particle in Mg-Al alloy melt, and *a*_*Al*_ is activity of Al particle in Mg-Al alloy melt.

Because C and Al_4_C_3_ exist in the form of solid particles in the Mg alloy melt, it is assumed that their activity is 1. The activity coefficient of Al in Mg-Al alloy can be expressed as follows [33]:

$$\log\gamma_{Al}=-1.02{\left(1‐X_{Al}\right)}^{2}+0.68{\left(1‐X_{Al}\right)}^{3}$$ (5)

where *γ*_Al_ is activity coefficient of Al_4_C_3_ particle in Mg-Al alloy melt, and *X*_*Al*_ is Mass percentage of Al in Mg-Al alloy.

**Fig 9:**
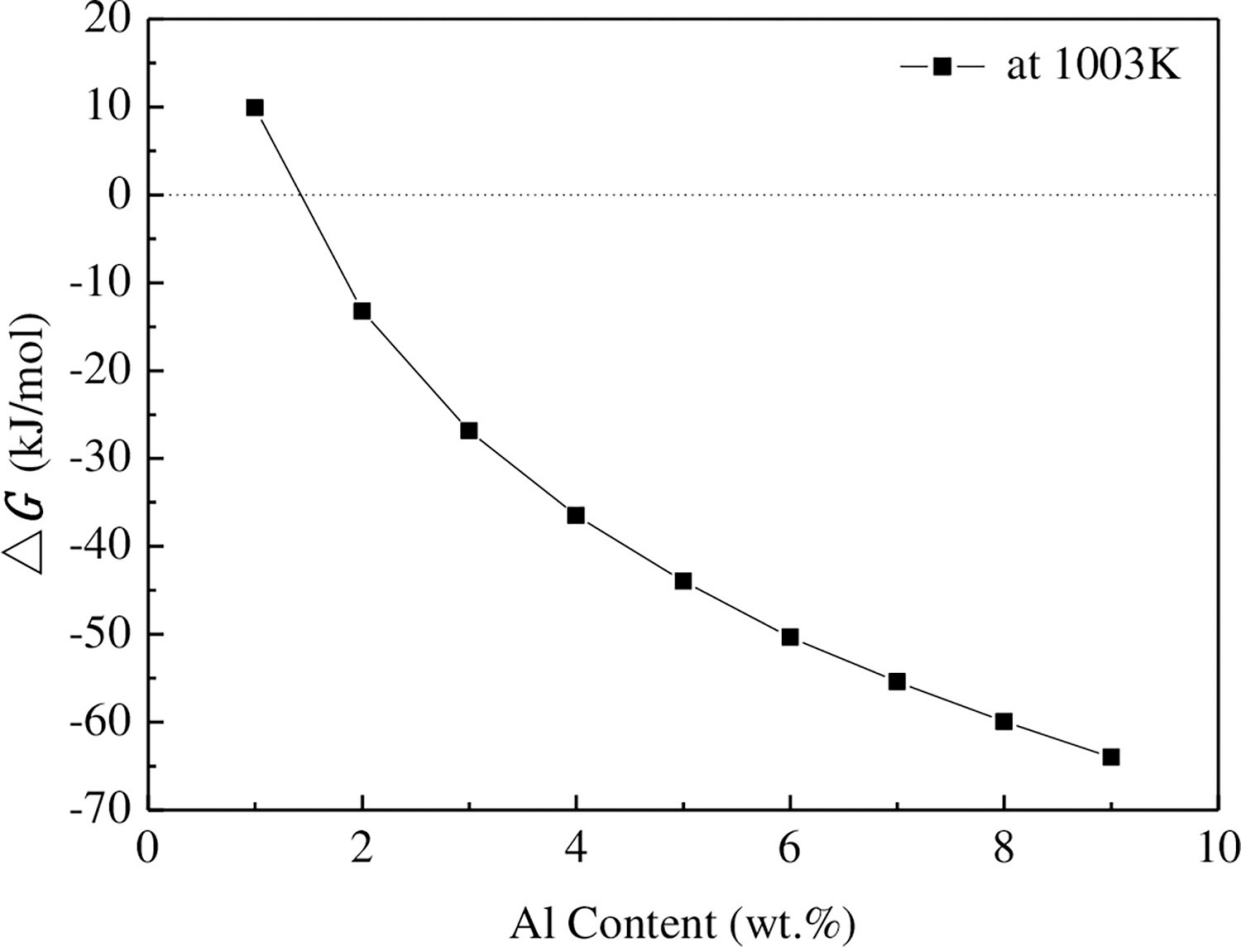
Variation in the Gibbs free energy of Al_4_C_3_ with Al content.

The Gibbs free energy for the formation of Al_4_C_3_ in Mg-Al alloys with different Al contents can be calculated by substituting Eq (5) into Eq (4), as shown in Fig 9.

It can be seen from Fig 9 (**S2 Table in S1 File**) that the Gibbs free energy of Al_4_C_3_ in the Mg-Al alloy when the Al content is equal to 1.34wt.% is 0. When the Al content is less than 1.34 wt.% in Mg-Al alloys, the Gibbs free energy of forming Al_4_C_3_ is greater than 0. In other words, when the Al content is less than 1.34 wt.%, the thermodynamic conditions for the formation of Al_4_C_3_ are not satisfied. When the Al content is more than 1.34 wt.%, the Gibbs free energy for the formation of Al_4_C_3_ is less than 0, and the Gibbs free energy gradually decreases with the increase in the Al content. The thermodynamic calculation results of Lu et al. [32] suggest that when the Al content is less than 1 wt.%, the thermodynamic condition to generate Al_4_C_3_ is not met, which is similar to the results of this study. The thermodynamic calculation results are also validated by the experimental results in Fig 4. For pure Mg and AZ11(containing 1 wt.% Al) alloy, C refinement is adopted, and Al_4_C_3_ is not generated in the melt. Therefore, C has no thinning effect on the Mg-Al alloy. When the content of Al is 2 wt.%, Al_4_C_3_ can be formed in the melt, so C can obviously refine the alloy, and with the increase in the Al content, Al_4_C_3_ is generated. The driving force of Mg-Al gradually increases, leading to an increase in the refinement efficiency of Mg-Al alloy. Gao et al. [34] showed that when the carbides are formed by Mg and C at a temperature lower than 1077°C, the Gibbs free energy of the chemical reaction is positive. Therefore, from the perspective of thermodynamics, the occurrence of the chemical reaction between Mg and C is extremely difficult in principle. Based on the above analysis, the possible heterogeneous nucleating particles can only be Al_4_C_3_.

### 5.2 Allogeneic nucleation crystallography conditions of Al_4_C_3_

The physical and crystallographic properties of Mg and Al_4_C_3_ are listed in Table 3. Al_4_C_3_ has a high melting point of 2475 K and has an oblique hexagonal structure. The lattice constant of the base plane is very close to that of the Mg base plane.

**Table 3:** Physical properties of Mg and Al_4_C_3_ [35].

Phase	Density (g/cm^3^)	Melting point (K)	Crystal structure		Crystal type
			*a* (nm)	*c* (nm)	
**α-Mg**	1.738	922	0.3209	0.5210	Closed hexagonal
**Al**_**4**_ **C**_**3**_	2.972	2475	0.3339	2.4996	Oblique hexagonal

**Fig 10:**
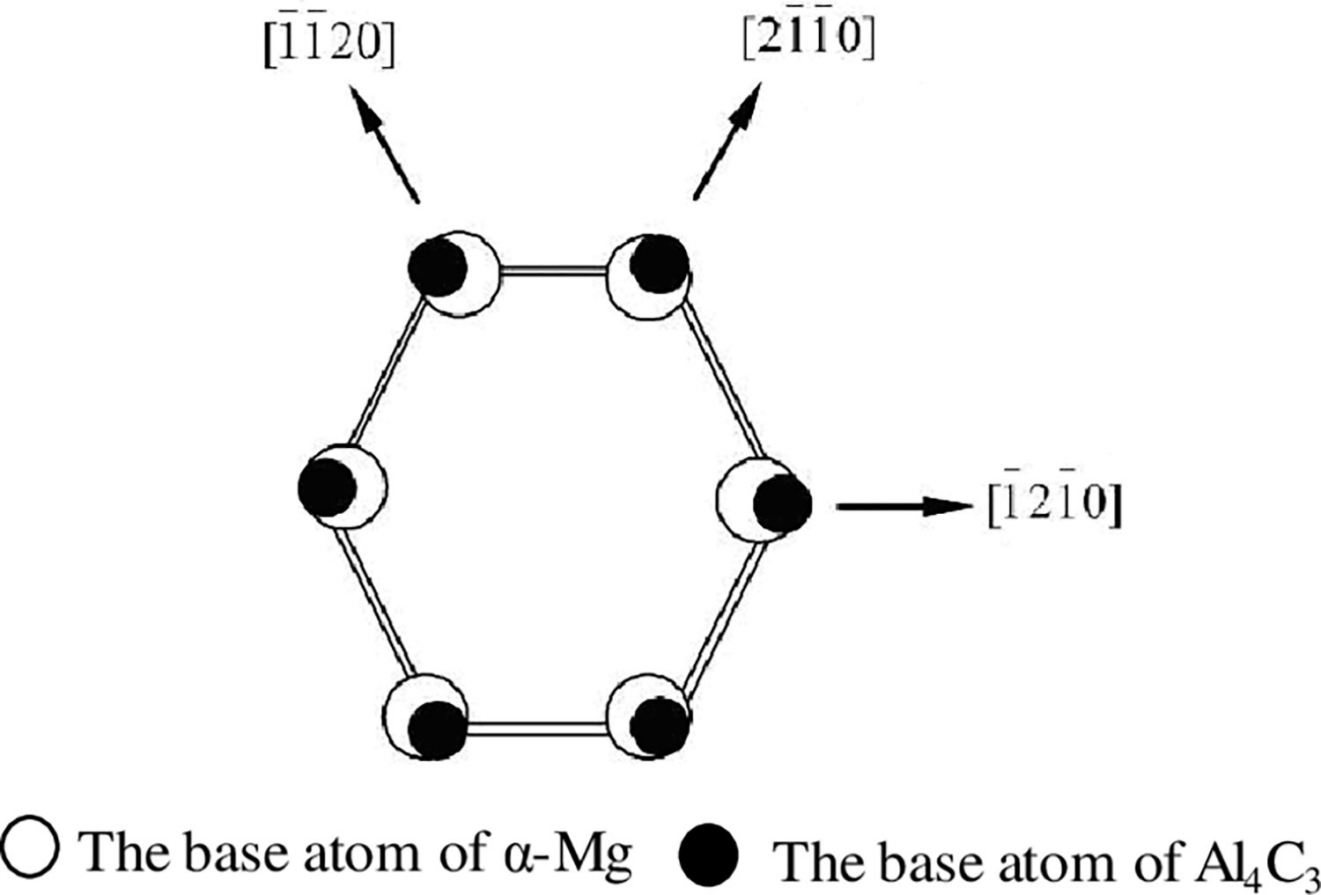
Scheme of the relationship of nucleation sites between α-Mg and Al_4_C_3_.

It is generally believed that the heterogeneous nucleation capacity value depends on the interfacial energy between the nucleation substrate and the crystalline phase. The factors affecting the interfacial energy mainly include the lattice mismatch between the substrate and the crystalline phase, surface morphology and chemical properties of the substrate, the electrostatic potential between the substrate and the crystalline phase. When the lattice mismatch causes a sharp increase in the elastic energy, the degree of mismatch is the main factor governing the interfacial energy. According to the interfacial coherence theory, a heterogeneous nucleating particle should have a higher binding force with the liquid atoms, i.e., the free energy of the two phases should be the minimum. This requires that the atomic arrangement and spacing of the surface atoms at the base plane are close to those of the new phase α-Mg. Bramfitt proposed to make the α-Mg crystal nucleus coincide with the low exponential surface of the heterogeneous nucleating particle to calculate the mismatch degree of the two phases. In this case, the mismatch degree can be expressed as follows [36]:

$$\delta_{(hkl)n}^{(hkl)s}=\sum_{i=1}^{3}\frac{\left|d_{{\left[uvw\right]}_{s}^{i}}\cos\theta -d_{{\left[uvw\right]}_{n}^{i}}\right|}{3d_{{\left[uvw\right]}_{n}^{i}}}$$ (6)

where the subscript *s* represents the nucleation substrate, the subscript *n* represents the nucleating crystal, (*hkl*) represents the low exponential plane, and [*uvw*] represents the three directions of this plane, and *θ* is the angle in the three directions of the low exponential plane. Fig 10 shows the relationship of the nucleation sites between α-Mg and Al_4_C_3_. It can be seen that nucleation occurs on the basal plane of C_3_ due to α-Mg and Al_4_C_3_. In the same hexagonal structure, the angle is 0 in the three directions. The mismatch degree between α-Mg and Al_4_C_3_ is calculated to be 4.05% according to the data in Table 1. When the mismatch of the two phases is less than 6%, Al_4_C_3_ can be used as an effective nucleating particle for α-Mg during the crystallization process [37].

### 5.3 Calculation of wetting angle

Let us assume that a spherical crown is formed on the surface of a heterogeneous nucleating particle, as shown in Fig 10. According to classical nucleation theory, three kinds of interfacial energies exist: *σ*_*LC*_, *σ*_*LS*_, and *σ*_*SC*_, which have the following relation when equilibrium is reached [38]:

$$\sigma_{LC}=\sigma_{SC}+\sigma_{LS}\cos\theta$$ (7)

where *σ*_*LC*_ is the interfacial energy between the melt and the nucleating particle (J/m^2^), *σ*_*SC*_ is the interfacial energy between the heterogenous nucleating particle and the crystal nucleus (J/m^2^), and *σ*_*LS*_ is interfacial energy between the crystal nucleus and liquid (J/m^2^). *θ* is wetting angle (°), which can be expressed as follows:

$$\cos\theta =\frac{\sigma_{LC}-\sigma_{SC}}{\sigma_{LS}}$$ (8)

**Fig 11:**
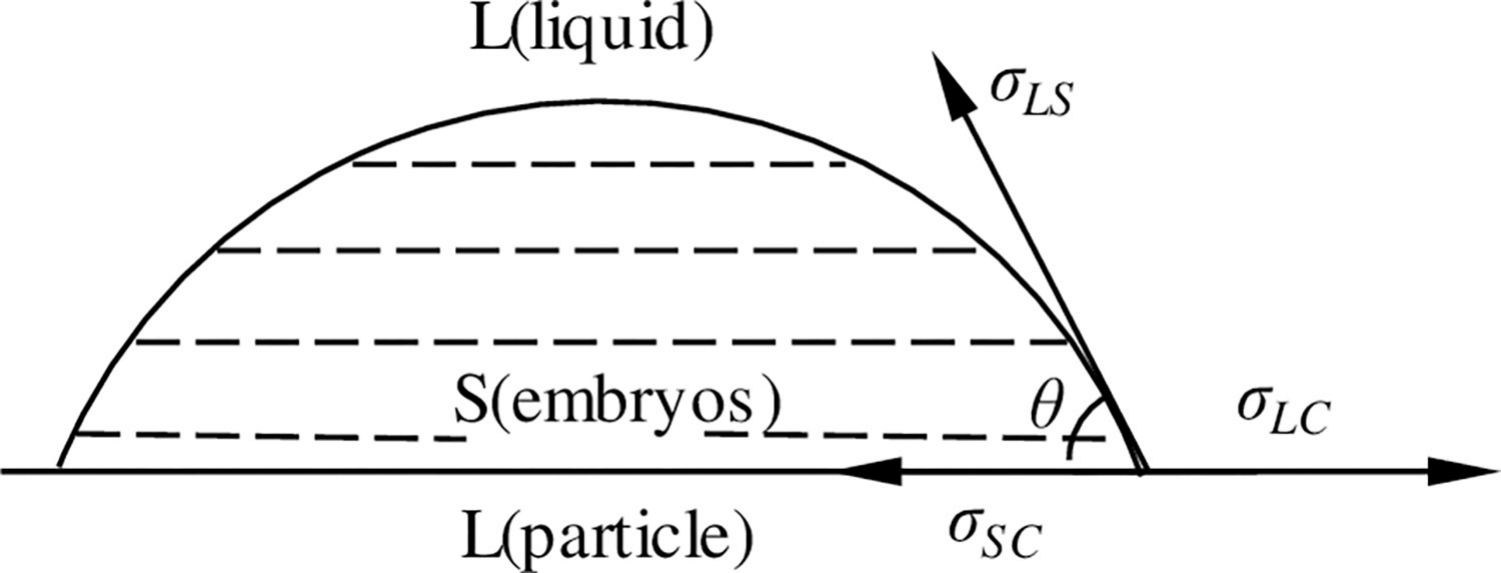
Scheme of heterogeneous nucleation.

Under the same degree of undercooling, the particles with smaller wetting angle relative to the matrix have a smaller nucleation barrier and are easier to nucleate, i.e., they have a stronger ability to refine the matrix. It can be seen from Eq (8) that to avoid negative value of cos*θ*, the value of *σ*_*SC*_ should be less than that of *σ*_*LC*_, and the smaller the value, the more obvious the thinning effect. This is because if *σ*_*SC*_ is small, the cos*θ* value is more likely to be close to 1, i.e., *θ* is closer to 0. In this case, the interfacial tension between nuclei and heterogeneous nucleation cytoplasmic points is smaller, which is more conducive to heterogeneous nucleation, as shown in Fig 11.

According to the above analysis, the wetting angle plays an important role in heterogeneous nucleation, and the value of wetting angle directly affects the difficulty of non-spontaneous nucleation during the thinning process. A small wetting angle requires a small critical nucleation radius, and non-spontaneous nucleation is easy to realize. Therefore, the refining effect of heterogeneous nucleating particles on the alloy melt can be more intuitively reflected if the value of wetting angle is determined. However, it is difficult to determine the wetting angle of α-Mg on Al_4_C_3_ by experiments due to the easy combustion of Mg alloy during the melting process. The results show that the addition of refiner can affect the degree of undercooling for nucleation [39]. In other words, different refiners have different abilities to refine the matrix. According to the classical nucleation theory, the smaller the wetting angle, the smaller the undercooling degree of nucleation. Therefore, we attempted to establish the relationship between the degree of undercooling and the wetting angle. Huang et al. [40] deduced the following mathematical model for the wetting angle based on thermodynamics and dynamics:

$$f(\theta )=\frac{\Delta G_{ne}^{2}T_{ne}}{\Delta G_{no}^{2}T_{no}}$$ (9)

where *T*_*ne*_ is the heterogenous nucleation temperature (K), *T*_*no*_ is the homogeneous nucleation temperature (K), Δ*G*_*ne*_ is the heterogeneous nucleation work (J), and Δ*G*_*no*_ is the homogeneous nucleation work (J).

*f*(*θ*) is also defined as follows [33]:

$$f(\theta )=\frac{1}{4}(2-3\cos\theta +\cos\theta^{3})$$ (10)

Hoffman derived the following approximate expressions for Δ*G*_*ne*_, and Δ*G*_*no*_ [41]:

$$\Delta G_{ne}=\frac{\Delta H_{m}\Delta T_{ne}}{T_{m}}\frac{T_{ne}}{T_{m}}$$ (11)

$$\Delta G_{no}=\frac{\Delta H_{m}\Delta T_{no}}{T_{m}}\frac{T_{no}}{T_{m}}$$ (12)

Substituting Eqs (11) and (13) into Eq (11), we get:

$$f(\theta )=\frac{\Delta T_{ne}^{2}T_{ne}^{3}}{\Delta T_{no}^{2}T_{no}^{3}}$$ (13)

Where Δ*T*_*ne*_ is the undercooling degree of heteronucleation (K), and Δ*T*_*no*_ is the undercooling degree of homogeneous nucleation (K).

**Fig 12:**
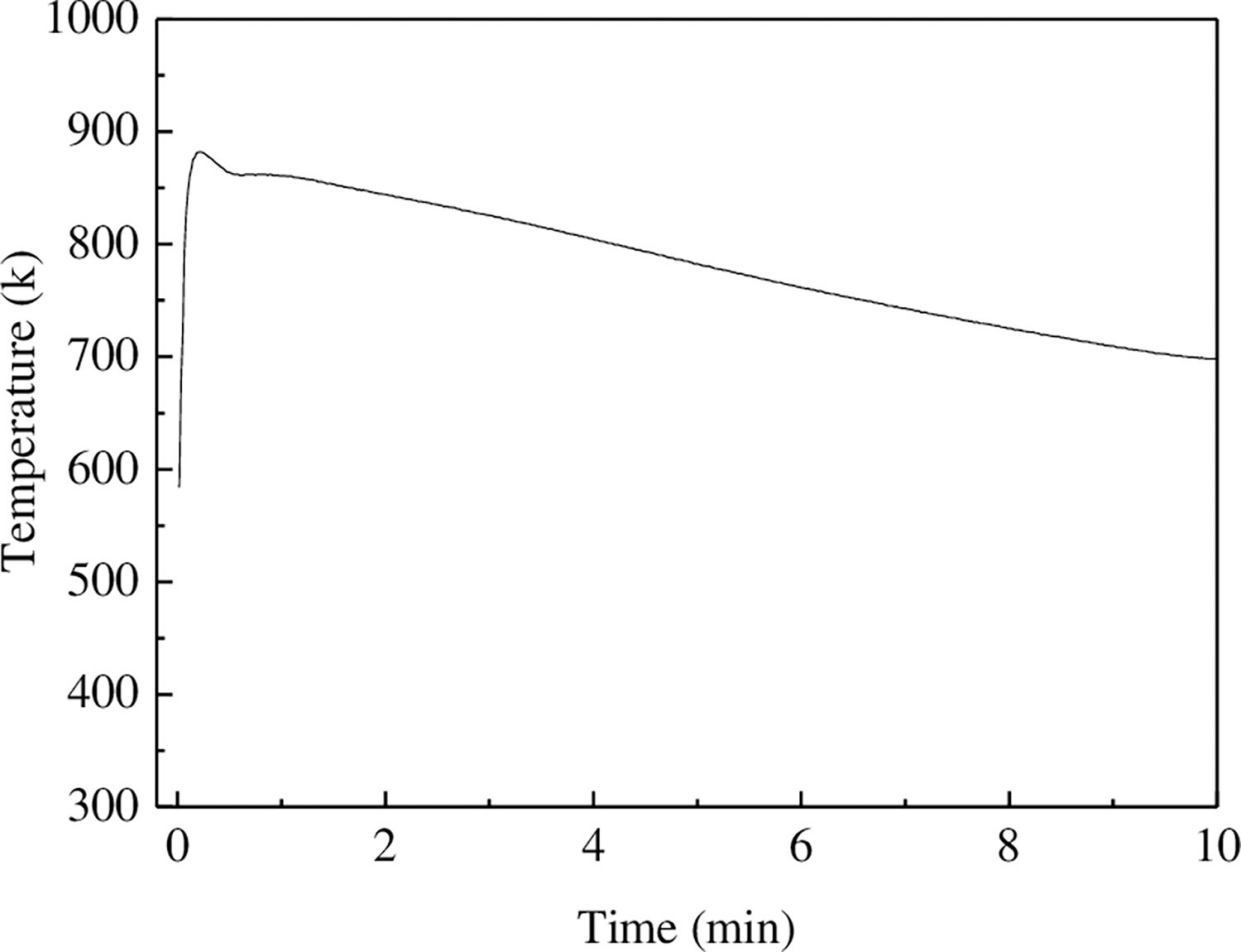
Solidification curve of AZ91 alloy.

The addition of Al_4_C_3_ increases the precipitation temperature of α-Mg, which indicates that Al_4_C_3_ acts as a heterogeneous nucleus. This causes the alloy to undergo the L → L+α-Mg transformation earlier and also leads to a decrease in the undercooling of the alloy. The undercooling curve appears when the undercooling is low. With the help of grain refiner, the nucleation rate and number of nucleants in the melt increase. Therefore, the melt does not need undercooling or fine grain structure under very small undercooling conditions. Based on the solidification curve of heterogeneous nucleation, the starting precipitation temperature of α-Mg during heterogeneous nucleation was measured and the theoretical crystallization temperature was compared to obtain the degree of undercooling in the heterogeneous nucleation. Fig 12 shows the measured solidification curve of AZ91 alloy after refinement.

It can be seen from the Fig 12 (**S3 Table in S1 File**) that the heteronucleation temperature is 862 K. Since there are few other elements in the AZ91 alloy, it is assumed that they have a minor influence on the solidification. Therefore, we approximate the solidification of Mg-9Al binary alloy instead of AZ91 alloy. It is clear from the phase diagram that the melting point of Mg-9Al alloy is 876 K [42], so Δ*T*_*ne*_ = 14 K. For alloys, the spontaneous nucleation temperature can be approximated by the following equation [40]:

$$\Delta T_{no}\approx 0.2T_{L}$$ (14)

Therefore, the undercooling degree of homogeneous nucleation of AZ91 alloy can be obtained as follows:

$$\Delta T_{no}\approx 0.2\times 876=175.2\;K$$

Substituting the expression of *T*_*ne*_, *T*_*no*_, Δ*T*_*ne*_, and Δ*T*_*no*_ in Eq (13), we get

f(θ)=0.00608 (15)

The wetting angle of Al_4_C_3_ can be obtained by substituting the obtained value of *f*(*θ*) into Eq (10). Accordingly, the wetting angle of Al_4_C_3_ in AZ91 alloy melt is calculated to be 24.3°.

## 6. Conclusion

In this study, the refinement effect and relevant mechanism of C on Mg-Al alloy were investigated through microstructural observation and theoretical calculation. The main results are summarized as follows:

1. C had no thinning effect on the Mg-Al alloy when the Al content was less than 1 wt.%. When the Al content was greater than 2%, the refining efficiency is 62%, and the refining effect increased with the increase in the Al content. When the Al content reached 9 wt.%, the refinement efficiency is 79%.
2. The presence of Al-C-O in the matrix was verified through microstructural observation. The presence of O was attributed to the hydrolysis during sample preparation, and the phase size was approximately 5 μm, which confirmed the presence of Al_4_C_3_ phase as a heterogeneous nucleating particle.
3. The theoretical calculations suggested that Al_4_C_3_ heterogeneous nucleating particle could not be generated when the Al content in the Mg alloy was less than 1.34%, which implied that there was no thinning effect under this case. The crystallographic calculations revealed that the mismatch between Al_4_C_3_ phase and Mg alloy matrix was only 4.05%, and Al_4_C_3_ particles could be regarded as the heterogeneous nucleating particles of α-Mg. By combining the measured solidification curves with the classical nucleation theory, the wetting angle of Mg-Al alloy on Al_4_C_3_ was calculated to be 24.3°.

## Supporting information

S1 File (DOCX)
