# Supplementary material for: Study on the grain refinement mechanism of Mg-Al alloy based on carbon addition
Source: PLoS One. 2022 Aug 4;17(8):e0271583. doi: 10.1371/journal.pone.0271583 (PMC9352047; doi:10.1371/journal.pone.0271583)
Supplement: S1 File — (DOCX) [file pone.0271583.s001.docx]

**S1 Table. Refinement parameter of Ar+CO_2_ in Mg-Al alloys with different Al contents**

| **Refinement state** | **Befor refinement**  **(μm)** | **After refinement**  **(μm)** | **Refinement efficiency**  **(%)** |
| --- | --- | --- | --- |
| **Pure Mg** | 2332 | 2309 | 0 |
| **AZ11** | 508 | 504 | 0 |
| **AZ21** | 413 | 157 | 62 |
| **AZ31** | 329 | 109 | 66.9 |
| **AZ61** | 311 | 98 | 68.8 |
| **AZ91** | 296 | 62.5 | 78.9 |

**S2 Table.** **Variation** **data in the Gibbs free energy of Al_4_C_3_ with Al content**

| **Al Content**  **(wt.%)** | **Gibbs free energy**  **(kJ/mol)** |
| --- | --- |
| 1 | 9.87678 |
| 2 | -13.26556 |
| 3 | -26.82659 |
| 4 | -36.47775 |
| 5 | -43.9877 |
| 6 | -50.36681 |
| 7 | -55.38613 |
| 8 | -59.95115 |
| 9 | -64.00198 |

**S3 Table.** **Measured data with temperature of AZ91 alloy after refinement**

| **Number** | **Time**  **（min）** | **Temperature**  **（K）** |
| --- | --- | --- |
| 1 | 0.01667 | 583.8 |
| 2 | 0.03333 | 677.3 |
| 3 | 0.05 | 724.7 |
| 4 | 0.06667 | 790.2 |
| 5 | 0.08333 | 826.7 |
| 6 | 0.1 | 847.7 |
| 7 | 0.11667 | 860.7 |
| 8 | 0.13333 | 868.1 |
| 9 | 0.15 | 875.4 |
| 10 | 0.16667 | 877.2 |
| 11 | 0.18333 | 881.1 |
| 12 | 0.2 | 881.7 |
| 13 | 0.21667 | 882.2 |
| 14 | 0.23333 | 881.7 |
| 15 | 0.25 | 881.1 |
| 16 | 0.26667 | 880 |
| 17 | 0.28333 | 878.8 |
| 18 | 0.3 | 877.7 |
| 19 | 0.31667 | 876.6 |
| 20 | 0.33333 | 875.4 |
| 21 | 0.35 | 873.7 |
| 22 | 0.36667 | 872.6 |
| 23 | 0.38333 | 871.5 |
| 24 | 0.4 | 870.3 |
| 25 | 0.41667 | 868.6 |
| 26 | 0.43333 | 867.5 |
| 27 | 0.45 | 866.4 |
| 28 | 0.46667 | 864.7 |
| 29 | 0.48333 | 864.1 |
| 30 | 0.5 | 863.6 |
| 31 | 0.51667 | 863 |
| 32 | 0.53333 | 862.4 |
| 33 | 0.55 | 861.9 |
| 34 | 0.56667 | 861.9 |
| 35 | 0.58333 | 861.9 |
| 36 | 0.6 | 861.3 |
| 37 | 0.61667 | 861.3 |
| 38 | 0.63333 | 861.3 |
| 39 | 0.65 | 861.8 |
| 40 | 0.66667 | 861.9 |
| 41 | 0.68333 | 861.9 |
| 42 | 0.7 | 861.9 |
| 43 | 0.71667 | 861.9 |
| 44 | 0.73333 | 861.9 |
| 45 | 0.75 | 861.3 |
| 46 | 0.76667 | 861.9 |
| 47 | 0.78333 | 861.8 |
| 48 | 0.8 | 861.9 |
| 49 | 0.81667 | 861.9 |
| 50 | 0.83333 | 861.9 |
| 51 | 0.85 | 861.8 |
| 52 | 0.86667 | 861.3 |
| 53 | 0.88333 | 861.9 |
| 54 | 0.9 | 861.9 |
| 55 | 0.91667 | 861.2 |
| 56 | 0.93333 | 861.3 |
| 57 | 0.95 | 861.3 |
| 58 | 0.96667 | 861.2 |
| 59 | 0.98333 | 860.7 |
| 60 | 1 | 860.7 |
| 61 | 1.01667 | 860.7 |
| 62 | 1.03333 | 860.7 |
| 63 | 1.05 | 860.2 |
| 64 | 1.06667 | 860.2 |
| 65 | 1.08333 | 860.2 |
| 66 | 1.1 | 860.2 |
| 67 | 1.11667 | 859.6 |
| 68 | 1.13333 | 859.6 |
| 69 | 1.15 | 859 |
| 70 | 1.16667 | 859 |
| 71 | 1.18333 | 858.5 |
| 72 | 1.2 | 858.4 |
| 73 | 1.21667 | 858.5 |
| 74 | 1.23333 | 857.8 |
| 75 | 1.25 | 857.9 |
| 76 | 1.26667 | 857.3 |
| 77 | 1.28333 | 857.3 |
| 78 | 1.3 | 857.3 |
| 79 | 1.31667 | 856.8 |
| 80 | 1.33333 | 856.8 |
| 81 | 1.35 | 855.6 |
| 82 | 1.36667 | 855.6 |
| 83 | 1.38333 | 855.6 |
| 84 | 1.4 | 855.1 |
| 85 | 1.41667 | 855.1 |
| 86 | 1.43333 | 853.9 |
| 87 | 1.45 | 853.9 |
| 88 | 1.46667 | 854 |
| 89 | 1.48333 | 853.4 |
| 90 | 1.5 | 853.4 |
| 91 | 1.51667 | 852.8 |
| 92 | 1.53333 | 852.2 |
| 93 | 1.55 | 852.2 |
| 94 | 1.56667 | 851.6 |
| 95 | 1.58333 | 851.6 |
| 96 | 1.6 | 851.1 |
| 97 | 1.61667 | 851.1 |
| 98 | 1.63333 | 850.5 |
| 99 | 1.65 | 850 |
| 100 | 1.66667 | 849.9 |
| 101 | 1.68333 | 850 |
| 102 | 1.7 | 849.4 |
| 103 | 1.71667 | 849.4 |
| 104 | 1.73333 | 848.2 |
| 105 | 1.75 | 848.8 |
| 106 | 1.76667 | 847.7 |
| 107 | 1.78333 | 848.3 |
| 108 | 1.8 | 847.7 |
| 109 | 1.81667 | 847.6 |
| 110 | 1.83333 | 847.1 |
| 111 | 1.85 | 846.5 |
| 112 | 1.86667 | 846.5 |
| 113 | 1.88333 | 846 |
| 114 | 1.9 | 845.4 |
| 115 | 1.91667 | 845.4 |
| 116 | 1.93333 | 844.8 |
| 117 | 1.95 | 844.8 |
| 118 | 1.96667 | 844.8 |
| 119 | 1.98333 | 844.2 |
| 120 | 2 | 844.3 |
| 121 | 2.01667 | 843.8 |
| 122 | 2.03333 | 843.7 |
| 123 | 2.05 | 843.1 |
| 124 | 2.06667 | 843.1 |
| 125 | 2.08333 | 842.5 |
| 126 | 2.1 | 842 |
| 127 | 2.11667 | 842 |
| 128 | 2.13333 | 842 |
| 129 | 2.15 | 841.4 |
| 130 | 2.16667 | 840.9 |
| 131 | 2.18333 | 840.8 |
| 132 | 2.2 | 840.3 |
| 133 | 2.21667 | 840.3 |
| 134 | 2.23333 | 839.7 |
| 135 | 2.25 | 839.7 |
| 136 | 2.26667 | 839.2 |
| 137 | 2.28333 | 839.2 |
| 138 | 2.3 | 838.6 |
| 139 | 2.31667 | 838 |
| 140 | 2.33333 | 838 |
| 141 | 2.35 | 838 |
| 142 | 2.36667 | 837.5 |
| 143 | 2.38333 | 837.5 |
| 144 | 2.4 | 836.8 |
| 145 | 2.41667 | 836.8 |
| 146 | 2.43333 | 836.3 |
| 147 | 2.45 | 835.7 |
| 148 | 2.46667 | 835.7 |
| 149 | 2.48333 | 835.2 |
| 150 | 2.5 | 835.2 |
| 151 | 2.51667 | 834.6 |
| 152 | 2.53333 | 834.6 |
| 153 | 2.55 | 834 |
| 154 | 2.56667 | 833.4 |
| 155 | 2.58333 | 833.5 |
| 156 | 2.6 | 833.4 |
| 157 | 2.61667 | 832.9 |
| 158 | 2.63333 | 833 |
| 159 | 2.65 | 832.3 |
| 160 | 2.66667 | 832.4 |
| 161 | 2.68333 | 831.8 |
| 162 | 2.7 | 831.2 |
| 163 | 2.71667 | 831.2 |
| 164 | 2.73333 | 830 |
| 165 | 2.75 | 830.1 |
| 166 | 2.76667 | 830.1 |
| 167 | 2.78333 | 829.5 |
| 168 | 2.8 | 829.5 |
| 169 | 2.81667 | 828.9 |
| 170 | 2.83333 | 828.9 |
| 171 | 2.85 | 828.4 |
| 172 | 2.86667 | 827.7 |
| 173 | 2.88333 | 827.7 |
| 174 | 2.9 | 827.2 |
| 175 | 2.91667 | 827.2 |
| 176 | 2.93333 | 826.6 |
| 177 | 2.95 | 826.7 |
| 178 | 2.96667 | 826.1 |
| 179 | 2.98333 | 826 |
| 180 | 3 | 825.5 |
| 181 | 3.01667 | 825.5 |
| 182 | 3.03333 | 824.9 |
| 183 | 3.05 | 824.4 |
| 184 | 3.06667 | 824.4 |
| 185 | 3.08333 | 823.8 |
| 186 | 3.1 | 823.8 |
| 187 | 3.11667 | 823.2 |
| 188 | 3.13333 | 822.6 |
| 189 | 3.15 | 822.1 |
| 190 | 3.16667 | 822.1 |
| 191 | 3.18333 | 822.1 |
| 192 | 3.2 | 821.5 |
| 193 | 3.21667 | 820.9 |
| 194 | 3.23333 | 821 |
| 195 | 3.25 | 820.3 |
| 196 | 3.26667 | 819.8 |
| 197 | 3.28333 | 819.8 |
| 198 | 3.3 | 819.2 |
| 199 | 3.31667 | 819.2 |
| 200 | 3.33333 | 818.7 |
| 201 | 3.35 | 818.7 |
| 202 | 3.36667 | 818.1 |
| 203 | 3.38333 | 818.1 |
| 204 | 3.4 | 817.5 |
| 205 | 3.41667 | 817 |
| 206 | 3.43333 | 817 |
| 207 | 3.45 | 816.4 |
| 208 | 3.46667 | 815.8 |
| 209 | 3.48333 | 815.8 |
| 210 | 3.5 | 815.3 |
| 211 | 3.51667 | 814.7 |
| 212 | 3.53333 | 814.7 |
| 213 | 3.55 | 814.1 |
| 214 | 3.56667 | 813.6 |
| 215 | 3.58333 | 813.6 |
| 216 | 3.6 | 813.5 |
| 217 | 3.61667 | 812.9 |
| 218 | 3.63333 | 812.4 |
| 219 | 3.65 | 811.8 |
| 220 | 3.66667 | 811.8 |
| 221 | 3.68333 | 811.2 |
| 222 | 3.7 | 811.3 |
| 223 | 3.71667 | 810.7 |
| 224 | 3.73333 | 810.1 |
| 225 | 3.75 | 809.6 |
| 226 | 3.76667 | 809.6 |
| 227 | 3.78333 | 808.9 |
| 228 | 3.8 | 809 |
| 229 | 3.81667 | 808.4 |
| 230 | 3.83333 | 808.4 |
| 231 | 3.85 | 807.8 |
| 232 | 3.86667 | 807.2 |
| 233 | 3.88333 | 807.3 |
| 234 | 3.9 | 806.7 |
| 235 | 3.91667 | 806.1 |
| 236 | 3.93333 | 806.1 |
| 237 | 3.95 | 805.6 |
| 238 | 3.96667 | 805 |
| 239 | 3.98333 | 805 |
| 240 | 4 | 804.4 |
| 241 | 4.01667 | 803.8 |
| 242 | 4.03333 | 803.8 |
| 243 | 4.05 | 803.3 |
| 244 | 4.06667 | 802.7 |
| 245 | 4.08333 | 802.7 |
| 246 | 4.1 | 802.1 |
| 247 | 4.11667 | 802.1 |
| 248 | 4.13333 | 801.5 |
| 249 | 4.15 | 801 |
| 250 | 4.16667 | 801 |
| 251 | 4.18333 | 800.4 |
| 252 | 4.2 | 799.9 |
| 253 | 4.21667 | 799.3 |
| 254 | 4.23333 | 799.9 |
| 255 | 4.25 | 798.7 |
| 256 | 4.26667 | 798.7 |
| 257 | 4.28333 | 798.1 |
| 258 | 4.3 | 798.1 |
| 259 | 4.31667 | 797.5 |
| 260 | 4.33333 | 797 |
| 261 | 4.35 | 797 |
| 262 | 4.36667 | 796.4 |
| 263 | 4.38333 | 796.4 |
| 264 | 4.4 | 795.3 |
| 265 | 4.41667 | 795.3 |
| 266 | 4.43333 | 794.7 |
| 267 | 4.45 | 794.7 |
| 268 | 4.46667 | 794.1 |
| 269 | 4.48333 | 793.6 |
| 270 | 4.5 | 793.6 |
| 271 | 4.51667 | 793 |
| 272 | 4.53333 | 793 |
| 273 | 4.55 | 792.4 |
| 274 | 4.56667 | 792.4 |
| 275 | 4.58333 | 791.8 |
| 276 | 4.6 | 791.3 |
| 277 | 4.61667 | 790.7 |
| 278 | 4.63333 | 790.1 |
| 279 | 4.65 | 790.1 |
| 280 | 4.66667 | 790.1 |
| 281 | 4.68333 | 789.5 |
| 282 | 4.7 | 789 |
| 283 | 4.71667 | 788.4 |
| 284 | 4.73333 | 788.4 |
| 285 | 4.75 | 787.8 |
| 286 | 4.76667 | 787.9 |
| 287 | 4.78333 | 786.7 |
| 288 | 4.8 | 786.7 |
| 289 | 4.81667 | 786.7 |
| 290 | 4.83333 | 786.1 |
| 291 | 4.85 | 786.2 |
| 292 | 4.86667 | 785.5 |
| 293 | 4.88333 | 785 |
| 294 | 4.9 | 784.9 |
| 295 | 4.91667 | 784.4 |
| 296 | 4.93333 | 783.8 |
| 297 | 4.95 | 783.8 |
| 298 | 4.96667 | 782.7 |
| 299 | 4.98333 | 782.7 |
| 300 | 5 | 782.7 |
| 301 | 5.01667 | 782.1 |
| 302 | 5.03333 | 781.6 |
| 303 | 5.05 | 780.9 |
| 304 | 5.06667 | 781 |
| 305 | 5.08333 | 780.4 |
| 306 | 5.1 | 780.4 |
| 307 | 5.11667 | 780.4 |
| 308 | 5.13333 | 779.8 |
| 309 | 5.15 | 779.3 |
| 310 | 5.16667 | 778.6 |
| 311 | 5.18333 | 778.6 |
| 312 | 5.2 | 778.1 |
| 313 | 5.21667 | 778.1 |
| 314 | 5.23333 | 777.5 |
| 315 | 5.25 | 776.9 |
| 316 | 5.26667 | 777 |
| 317 | 5.28333 | 776.4 |
| 318 | 5.3 | 776.4 |
| 319 | 5.31667 | 775.8 |
| 320 | 5.33333 | 775.2 |
| 321 | 5.35 | 775.3 |
| 322 | 5.36667 | 774.6 |
| 323 | 5.38333 | 774.1 |
| 324 | 5.4 | 774.1 |
| 325 | 5.41667 | 774.1 |
| 326 | 5.43333 | 773.5 |
| 327 | 5.45 | 772.9 |
| 328 | 5.46667 | 772.3 |
| 329 | 5.48333 | 772.4 |
| 330 | 5.5 | 771.8 |
| 331 | 5.51667 | 771.8 |
| 332 | 5.53333 | 771.2 |
| 333 | 5.55 | 771.2 |
| 334 | 5.56667 | 770.7 |
| 335 | 5.58333 | 770.1 |
| 336 | 5.6 | 769.5 |
| 337 | 5.61667 | 769.5 |
| 338 | 5.63333 | 768.9 |
| 339 | 5.65 | 768.9 |
| 340 | 5.66667 | 768.3 |
| 341 | 5.68333 | 768.3 |
| 342 | 5.7 | 767.8 |
| 343 | 5.71667 | 767.2 |
| 344 | 5.73333 | 766.6 |
| 345 | 5.75 | 766.6 |
| 346 | 5.76667 | 766 |
| 347 | 5.78333 | 766.1 |
| 348 | 5.8 | 766 |
| 349 | 5.81667 | 765.5 |
| 350 | 5.83333 | 764.9 |
| 351 | 5.85 | 764.4 |
| 352 | 5.86667 | 764.3 |
| 353 | 5.88333 | 763.8 |
| 354 | 5.9 | 763.8 |
| 355 | 5.91667 | 763.2 |
| 356 | 5.93333 | 763.2 |
| 357 | 5.95 | 762.6 |
| 358 | 5.96667 | 762 |
| 359 | 5.98333 | 762 |
| 360 | 6 | 761.4 |
| 361 | 6.01667 | 761.4 |
| 362 | 6.03333 | 760.9 |
| 363 | 6.05 | 760.3 |
| 364 | 6.06667 | 760.9 |
| 365 | 6.08333 | 759.8 |
| 366 | 6.1 | 759.7 |
| 367 | 6.11667 | 759.7 |
| 368 | 6.13333 | 759.1 |
| 369 | 6.15 | 758.6 |
| 370 | 6.16667 | 758 |
| 371 | 6.18333 | 758.6 |
| 372 | 6.2 | 758 |
| 373 | 6.21667 | 757.4 |
| 374 | 6.23333 | 756.9 |
| 375 | 6.25 | 756.9 |
| 376 | 6.26667 | 756.3 |
| 377 | 6.28333 | 755.7 |
| 378 | 6.3 | 755.7 |
| 379 | 6.31667 | 755.7 |
| 380 | 6.33333 | 755.1 |
| 381 | 6.35 | 755.2 |
| 382 | 6.36667 | 754.6 |
| 383 | 6.38333 | 754 |
| 384 | 6.4 | 754 |
| 385 | 6.41667 | 753.4 |
| 386 | 6.43333 | 753.4 |
| 387 | 6.45 | 752.9 |
| 388 | 6.46667 | 752.8 |
| 389 | 6.48333 | 752.3 |
| 390 | 6.5 | 752.3 |
| 391 | 6.51667 | 751.7 |
| 392 | 6.53333 | 751.1 |
| 393 | 6.55 | 751.2 |
| 394 | 6.56667 | 750.6 |
| 395 | 6.58333 | 750.5 |
| 396 | 6.6 | 750 |
| 397 | 6.61667 | 750 |
| 398 | 6.63333 | 749.4 |
| 399 | 6.65 | 749.4 |
| 400 | 6.66667 | 749.4 |
| 401 | 6.68333 | 748.8 |
| 402 | 6.7 | 748.2 |
| 403 | 6.71667 | 748.2 |
| 404 | 6.73333 | 747.7 |
| 405 | 6.75 | 747.2 |
| 406 | 6.76667 | 747.1 |
| 407 | 6.78333 | 747.1 |
| 408 | 6.8 | 746 |
| 409 | 6.81667 | 746 |
| 410 | 6.83333 | 745.4 |
| 411 | 6.85 | 745.4 |
| 412 | 6.86667 | 745.4 |
| 413 | 6.88333 | 744.8 |
| 414 | 6.9 | 744.2 |
| 415 | 6.91667 | 744.8 |
| 416 | 6.93333 | 743.6 |
| 417 | 6.95 | 743.6 |
| 418 | 6.96667 | 743.1 |
| 419 | 6.98333 | 743.1 |
| 420 | 7 | 743.1 |
| 421 | 7.01667 | 742.5 |
| 422 | 7.03333 | 742.5 |
| 423 | 7.05 | 741.9 |
| 424 | 7.06667 | 741.9 |
| 425 | 7.08333 | 741.3 |
| 426 | 7.1 | 740.7 |
| 427 | 7.11667 | 740.7 |
| 428 | 7.13333 | 740.7 |
| 429 | 7.15 | 739.6 |
| 430 | 7.16667 | 739.6 |
| 431 | 7.18333 | 739.6 |
| 432 | 7.2 | 739.6 |
| 433 | 7.21667 | 738.5 |
| 434 | 7.23333 | 738.4 |
| 435 | 7.25 | 738.4 |
| 436 | 7.26667 | 737.9 |
| 437 | 7.28333 | 737.3 |
| 438 | 7.3 | 737.3 |
| 439 | 7.31667 | 736.7 |
| 440 | 7.33333 | 736.7 |
| 441 | 7.35 | 736.8 |
| 442 | 7.36667 | 736.1 |
| 443 | 7.38333 | 736.1 |
| 444 | 7.4 | 735.6 |
| 445 | 7.41667 | 735 |
| 446 | 7.43333 | 735 |
| 447 | 7.45 | 734.4 |
| 448 | 7.46667 | 734.4 |
| 449 | 7.48333 | 734.4 |
| 450 | 7.5 | 733.8 |
| 451 | 7.51667 | 733.8 |
| 452 | 7.53333 | 733.3 |
| 453 | 7.55 | 732.7 |
| 454 | 7.56667 | 732.7 |
| 455 | 7.58333 | 732.1 |
| 456 | 7.6 | 732.2 |
| 457 | 7.61667 | 731.5 |
| 458 | 7.63333 | 731.6 |
| 459 | 7.65 | 731 |
| 460 | 7.66667 | 730.4 |
| 461 | 7.68333 | 731 |
| 462 | 7.7 | 730.4 |
| 463 | 7.71667 | 729.8 |
| 464 | 7.73333 | 729.8 |
| 465 | 7.75 | 729.8 |
| 466 | 7.76667 | 729.2 |
| 467 | 7.78333 | 729.2 |
| 468 | 7.8 | 728.7 |
| 469 | 7.81667 | 728.7 |
| 470 | 7.83333 | 728.1 |
| 471 | 7.85 | 728.1 |
| 472 | 7.86667 | 727.5 |
| 473 | 7.88333 | 727.5 |
| 474 | 7.9 | 727.5 |
| 475 | 7.91667 | 726.3 |
| 476 | 7.93333 | 726.4 |
| 477 | 7.95 | 725.8 |
| 478 | 7.96667 | 725.8 |
| 479 | 7.98333 | 725.2 |
| 480 | 8 | 725.2 |
| 481 | 8.01667 | 725.2 |
| 482 | 8.03333 | 724.6 |
| 483 | 8.05 | 724.1 |
| 484 | 8.06667 | 724.1 |
| 485 | 8.08333 | 724 |
| 486 | 8.1 | 723.5 |
| 487 | 8.11667 | 723.5 |
| 488 | 8.13333 | 722.9 |
| 489 | 8.15 | 722.9 |
| 490 | 8.16667 | 722.3 |
| 491 | 8.18333 | 722.3 |
| 492 | 8.2 | 721.7 |
| 493 | 8.21667 | 721.8 |
| 494 | 8.23333 | 721.8 |
| 495 | 8.25 | 721.2 |
| 496 | 8.26667 | 721.2 |
| 497 | 8.28333 | 720.6 |
| 498 | 8.3 | 720 |
| 499 | 8.31667 | 719.4 |
| 500 | 8.33333 | 720 |
| 501 | 8.35 | 719.5 |
| 502 | 8.36667 | 719.4 |
| 503 | 8.38333 | 718.8 |
| 504 | 8.4 | 718.8 |
| 505 | 8.41667 | 718.3 |
| 506 | 8.43333 | 718.3 |
| 507 | 8.45 | 717.7 |
| 508 | 8.46667 | 717.7 |
| 509 | 8.48333 | 717.7 |
| 510 | 8.5 | 717.1 |
| 511 | 8.51667 | 717.2 |
| 512 | 8.53333 | 716.6 |
| 513 | 8.55 | 716.5 |
| 514 | 8.56667 | 716 |
| 515 | 8.58333 | 716 |
| 516 | 8.6 | 715.4 |
| 517 | 8.61667 | 715.4 |
| 518 | 8.63333 | 714.8 |
| 519 | 8.65 | 714.8 |
| 520 | 8.66667 | 714.3 |
| 521 | 8.68333 | 714.3 |
| 522 | 8.7 | 713.6 |
| 523 | 8.71667 | 713.6 |
| 524 | 8.73333 | 713.6 |
| 525 | 8.75 | 713.1 |
| 526 | 8.76667 | 713.1 |
| 527 | 8.78333 | 712.5 |
| 528 | 8.8 | 712.5 |
| 529 | 8.81667 | 712 |
| 530 | 8.83333 | 711.9 |
| 531 | 8.85 | 711.3 |
| 532 | 8.86667 | 711.4 |
| 533 | 8.88333 | 710.8 |
| 534 | 8.9 | 711.4 |
| 535 | 8.91667 | 710.2 |
| 536 | 8.93333 | 710.2 |
| 537 | 8.95 | 710.2 |
| 538 | 8.96667 | 709.6 |
| 539 | 8.98333 | 709.6 |
| 540 | 9 | 709.6 |
| 541 | 9.01667 | 709 |
| 542 | 9.03333 | 708.4 |
| 543 | 9.05 | 708.4 |
| 544 | 9.06667 | 708.4 |
| 545 | 9.08333 | 707.9 |
| 546 | 9.1 | 707.9 |
| 547 | 9.11667 | 707.9 |
| 548 | 9.13333 | 707.3 |
| 549 | 9.15 | 706.7 |
| 550 | 9.16667 | 706.7 |
| 551 | 9.18333 | 706.7 |
| 552 | 9.2 | 706.1 |
| 553 | 9.21667 | 706.2 |
| 554 | 9.23333 | 706.1 |
| 555 | 9.25 | 705.6 |
| 556 | 9.26667 | 705.6 |
| 557 | 9.28333 | 705 |
| 558 | 9.3 | 705 |
| 559 | 9.31667 | 705 |
| 560 | 9.33333 | 704.4 |
| 561 | 9.35 | 704.4 |
| 562 | 9.36667 | 703.8 |
| 563 | 9.38333 | 703.8 |
| 564 | 9.4 | 703.2 |
| 565 | 9.41667 | 703.2 |
| 566 | 9.43333 | 703.2 |
| 567 | 9.45 | 703.2 |
| 568 | 9.46667 | 702.7 |
| 569 | 9.48333 | 702.7 |
| 570 | 9.5 | 702.7 |
| 571 | 9.51667 | 702.1 |
| 572 | 9.53333 | 702.1 |
| 573 | 9.55 | 701.5 |
| 574 | 9.56667 | 701.5 |
| 575 | 9.58333 | 701.5 |
| 576 | 9.6 | 700.9 |
| 577 | 9.61667 | 700.9 |
| 578 | 9.63333 | 700.9 |
| 579 | 9.65 | 700.9 |
| 580 | 9.66667 | 700.3 |
| 581 | 9.68333 | 700.3 |
| 582 | 9.7 | 700.3 |
| 583 | 9.71667 | 699.8 |
| 584 | 9.73333 | 699.8 |
| 585 | 9.75 | 699.8 |
| 586 | 9.76667 | 699.8 |
| 587 | 9.78333 | 699.2 |
| 588 | 9.8 | 699.2 |
| 589 | 9.81667 | 699.2 |
| 590 | 9.83333 | 698.6 |
| 591 | 9.85 | 699.2 |
| 592 | 9.86667 | 698.6 |
| 593 | 9.88333 | 698.6 |
| 594 | 9.9 | 698 |
| 595 | 9.91667 | 698 |
| 596 | 9.93333 | 698 |
| 597 | 9.95 | 698 |
| 598 | 9.96667 | 698 |
| 599 | 9.98333 | 698 |
| 600 | 10 | 698 |
